# The Study of Intramuscular Nerve Distribution Patterns and Relative Spindle Abundance of the Thenar and Hypothenar Muscles in Human Hand

**DOI:** 10.1371/journal.pone.0051538

**Published:** 2012-12-10

**Authors:** Peng Xie, Yanjun Jiang, Xiaoming Zhang, Shengbo Yang

**Affiliations:** 1 Department of Anatomy, Zunyi Medical College, Zunyi, Guizhou, People's Republic of China; 2 Department of Anatomy and Cell Biology, University of Kansas Medical Center, Kansas City, Kansas, United States of America; Cinvestav-IPN, Mexico

## Abstract

**Background:**

The intramuscular nerve distribution and relative spindle abundance of the human hand have not been well defined, although this is important in guiding hand surgery.

**Methods:**

Forty human hands were dissected and subjected to modified Sihler’s stain and haematoxylin and eosin stain to investigate intramuscular nerve distribution and relative spindle abundance, respectively.

**Results:**

The flexor pollicis brevis (FPB), adductor pollicis (AP), and abductor digiti minimi (ADM) contain separate nerve compartments. Neural anastomoses were observed in the thenar and hypothenar muscles, including the Y-like, O-like, H-like, and U-like appearance. We found that U-like neural anastomoses may be the characteristic of the opponens muscles. The relative spindle abundance was the greatest in the opponens muscles which may coordinate fine movements.

**Conclusion:**

Except for the two opponens muscles, the rest of the thenar and hypothenar muscles could be used as whole muscle or half-muscle donors for muscle transplant. Our nerve map of the hand offers valuable guidance for hand reconstruction.

## Introduction

Although the origins and the precise courses of the nerves outside the thenar and hypothenar muscles have been morphologically documented using gross anatomy and microdissection methods [1]–[4], the precise intramuscular nerve distribution patterns of the thenar and hypothenar muscles have not been clearly described. Gross anatomy and microdissection have been the most commonly used methods, however, it should be noted that there are limitations when tracing nerve fibers from extramuscular branches to intramuscular terminal branches due to damaged muscle fibers and normal anatomical relationship between muscle cells and nerve fibers [5]–[6]. The precision should be gained from intramuscular nerve distribution studies of these hand muscles.

Muscle spindles are stretch receptors that are distributed within muscles and perceive the muscle length and the velocity of muscle length change [7]–[8]. Muscle spindles provide fundamental signals to the nervous system to control muscle tone, sense muscle position, regulate fine movement, exercise motor learning, and build plasticity of motor behaviors [9]–[10]. The density of spindles varies in different muscles of the same individual and also differs within the same muscle of different individuals [11]–[12]. Previous studies have found that muscle spindle densities are higher in flexor and deep muscles than that in extensor and superficial muscles [13]–[15]. However, until recently, Banks [16] suggested that the spindle density calculated from the number of spindles g^−1^ of muscle as a measure of relative spindle abundance is not correct, the relative spindle abundance of different muscles should be calculated using allometric analysis. The relative spindle abundance of the human hand has not been completely defined although some intrinsic hand muscles have been examined.

The thumb is the most important finger. It executes 40% of the functions of the hand while opposition executes 60% function of the thumb. Surgeons have attempted to help patients who have lost their thumbs, or other fingers, to recover functions and appearance. However, intrinsic hand muscles have not been well protected and utilized during clinical hand surgery so that some patients were left with hands that cannot function as expected. For this reason, it is necessary to study the intramuscular nerve distribution patterns and relative spindle abundance of the thenar and hypothenar muscles. Our study is intended to provide valuable data to anatomists and hand surgeons in several aspects such as to understand the adjustment of finger’s fine movement, to select the right tissue in hand surgery, to match correct tissues during neuromuscular transplant, and to inject blocking agents at right locations during anesthesia.

## Materials and Methods

### Subjects

The study was approved by Zunyi Medical College, Human Subject Study Committee. All cadavers were from persons who donated their bodies for education and research. Prior to the donation, written consents from persons and families and ethical approval from the Human Subject Study Committee were obtained. Twenty adult human cadavers (40 hands) were collected at Zunyi Medical College from 2008 to 2011, including 8 females and 12 males with an age-range of 56 to 82 years old. The cadavers were routinely fixed in formalin for two years. None of the donative cadavers revealed any evidence of significant pathology, surgical procedures or traumatic lesions to the hand or wrist.

### Gross Anatomical Observations

The thenar and hypothenar muscles’ origins and insertions, the spread of muscle fibers, and the extramuscular nerves branches were carefully dissected. Digital calipers (Mitutoyo, Kawasaki City, Japan) were used to locate the entry points of nerves and to measure the length of main extramuscular nerve branches.

### Modified Sihler’s Stain

The Sihler’s staining technique [17] was used with slight modifications: the muscles were washed and immersed in 3% KOH solution for maceration and depigmentation. For depigmentation, hydrogen peroxide (3%, 0.4 ml per 100 ml KOH) was added. It took 3 to 5 weeks for the specimen to be translucent.

The muscles were then washed with tap water for 1 hr and placed in Sihler’s Solution-I for 3–5 weeks. The decalcified muscles were washed again and stained with Sihler’s Solution-II. Total time for staining varied with the thickness of the muscles. The end point was when all nerves within the muscle were stained dark violet blue and the finest twigs could be seen under a dissecting microscope.

The stained muscles were washed and destained in Sihler’s Solution-I. After destaining, the muscles were neutralized with freshly made 0.05% lithium carbonate solution for 1–2 hours. The well-neutralized muscles were washed and transferred to graded glycerin solutions (40, 60 and 80%, respectively, each for 3–5 days). The cleared muscles were preserved in 100% glycerin with a few thymol crystals for transparency. Fascias around the muscles were carefully removed under a stereomicroscope. The superficial and deep surfaces of the muscles were photographed and the distributions of the intramuscular nerve branches in every muscle were drawn.

### Histological Examination

The thenar and hypothenar muscles were excised from their origins and marked with indelible ink so that the origin could be readily identified. The wet-muscle weight was recorded. Muscles were processed to standard histology and 10 µm was sectioned and stained using haematoxylin and eosin and then examined under light microscopes (Olympus Optical Company, Ltd., Tokyo, Japan). In our experiment, it is stipulated that the same muscle spindle appearing in serial sections was counted once. The muscle spindle was recorded when it first appears or disappears. In this case, it ensures accurate muscle spindle counts and removes errors associated with duplicate counts of the same muscle spindle.

The relative spindle abundance for each muscle was calculated according to the allometric analysis method developed by Banks [16]. This method was described in detail in his paper. Briefly, the relative spindle abundance for each muscle was calculated by dividing the actual number of spindles by the predicted number with correction. The corrected predicted number was calculated using the formula (*S*_pn_ = 20.5*m*_n_^0.49^) and the Snowdon’s ratio estimator.

### Statistical Analysis

Data were analyzed with one-way analysis of variance, P<0.05 is considered as significant.

## Results

### Extramuscular Nerve Course and Measurement

The recurrent branch of the median nerve enters abductor pollicis brevis (APB), opponens pollicis (OP), and FPB from the medial side at proximal 1/3 part of the muscles. The length of branches are listed Table 1. The ulnar nerve divides into superficial and deep branches between the pisiform and the hamate bones. The deep branch divides below the hypothenar fibro-muscular tunnel, traverses across the deep palm, and then divides into four terminal branches to ADM, flexor digiti minimi brevis (FDMB), opponens digiti minimi (ODM) and AP.

**Table 1:** Relative spindle abundance and extramuscular nerve trunk length of the thenar and hypothenar muscles.

Muscle	n = 20	mass (g)	Actual no.	Predicted no.With correction	Relative abundance	Extramuscular nerve trunklength (cm)
APB		7.73	37.5	67.01	0.56	2.29±0.22
FPB		6.86	39	63.20	0.62	1.70±0.12
OP		8.20	45	68.98	0.65	0.93±0.16
AP		10.93	41	79.41	0.52	0.72±0.19
ADM		6.68	25	62.38	0.40	1.64±0.20
FDMB		3.14	22	43.10	0.51	0.65±0.17
ODM		4.32	29.5	50.39	0.59	0.49±0.26

In 32 of the 40 hands (80%), the deep head of abductor pollicis brevis (dAPB) is innervated by a branch from the ulnar nerve, which enters the muscle from the proximal part.The average length of this branch is 2.23±0.6 cm. In 33 of the 40 hands (82.5%), a terminal branch of the ulnar nerve travels across the dAPB and enters the OP at its distal 1/3. The average length of this branch is 2.36±0.3 cm.

### Intramuscular Nerve Distribution

#### APB (Fig. 1)

A branch (marked as NT) of the recurrent branch of the median nerve enters APB from ulnar side at the junction between proximal third and middle third and divides into two major branches. One major branch travels to the ulnar part and the other to the radial portion. During its course toward the radial border, the main branch sends out 5 to 6 major branches and is curved to the direction of muscle insertion. Two major branches sub-divide into many minor branches densely distributed along the medial and lateral border at the junction of proximal third and middle third. Neural anastomoses are visible between minor branches.

**Figure 1:**
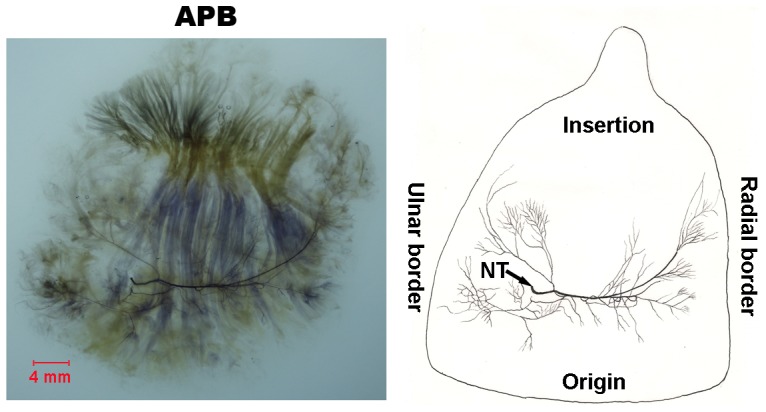
Intramuscular nerve distribution in the APB (right, deep side), drawing demonstrating several intramuscular branches arising from the branch of the recurrent nerve of median nerve (NT, nerve trunk). The scale bar represents 4 mm.

#### FPB (Fig. 2)

The superficial head of this muscle is supplied by the recurrent branch of the median nerve (marked as MNB) and the deep head by the deep branch of the ulnar nerve (marked as UNB). The nerve branch to the superficial head splits into 2 major branches after entering. During their course through the muscle, they divide into minor branches like a ramified tree. Neural anastomoses are observed between 2 major branches at the distal 2/3, and between minor branches with H-like, Y-like, polygon-like or O-like appearance. The deep head branch divides into 3–5 major branches after entering the muscle. Some course in parallel to the muscle’s longitudinal axis, and further sub-divide into minor branches toward the insertion point, the superficial side, and the deep side of the muscle. Neural anastomoses are observed between these minor branches. The remaining major branches from the ulnar nerve are distributed to the origin of the muscle and no anastomoses are observed.

#### OP (Fig. 3)

The OP receives double innervation by the recurrent branch of the median nerve and the deep branch of the ulnar nerve. The median nerve enters the muscle at the fusion point of proximal third and middle third from the radial side, and divides into 3 major branches. The thickest major branch extends to the ulnar border and the distal region of the muscle. During its course through the muscle, it sub-divides into minor branches with Y-like and H-like neural anastomoses. The second major branch is thinner and arises from the origin of the muscle. It spreads to the origin and the ulnar border of the muscle and sub-divides into minor branches with U-like and H-like anastomoses. Furthermore, these minor branches anastomose with the thickest major branch and with the branches from the ulnar nerve to make an O-like or Y-like appearance. The third major branch sub-divides into minor branches and is distributed to the origin and radial border of the muscle with O-like, Y-like and H-like anastomoses. This major branch continues as a small nerve branch and courses to the radial border of the muscle where it arches to the ulnar border at the fusion point of middle third and distal third. Anastomoses between the terminal branches and the branches of the deep branch of ulnar nerve are U-like loops.

**Figure 2:**
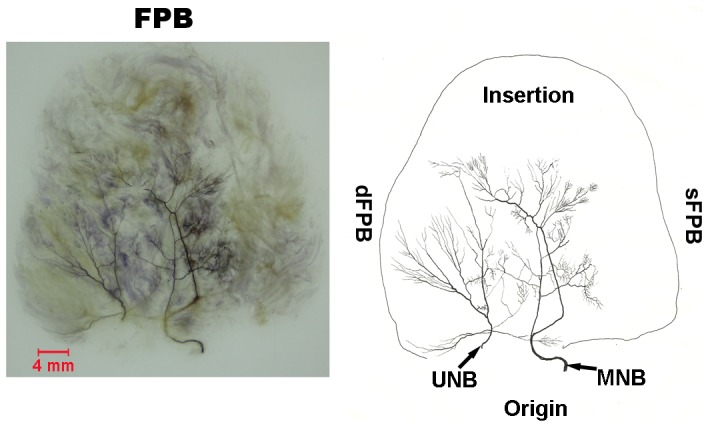
Intramuscular nerve distribution in the FPB (right, deep side), drawing demonstrating the sFPB was supplied by the branch of the median nerve and the dFPB was supplied by the branch of the ulnar nerve (MNB, branch of the median nerve; UNB, branch of the ulnar nerve). The scale bar represents 4 mm.

The deep branch of the ulnar nerve enters the muscle at the fusion point of proximal third and middle third from the ulnar side. One major branch is formed after entry and returns to the direction of muscle origin. During its course through the muscle, it sub-divides into minor branches. These minor branches anastomose with the thickest major branch from the median nerve in Y-like appearance.

**Figure 3:**
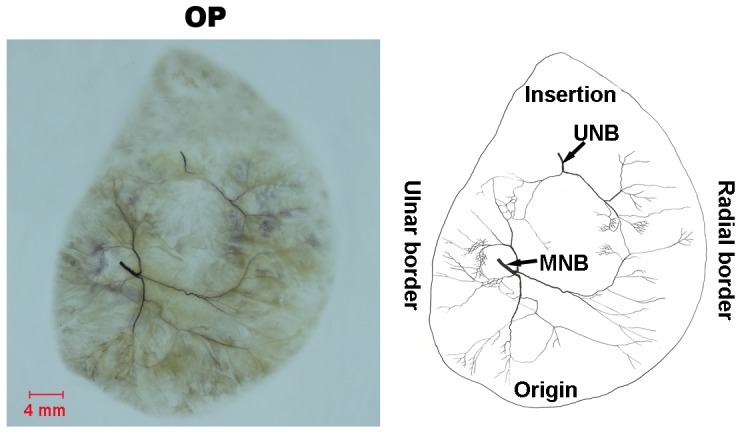
Intramuscular nerve distribution in the OP (right, deep side), drawing demonstrating the branching pattern of the branch of the median nerve and the branch of the ulnar nerve (MNB, branch of the median nerve; UNB, branch of the ulnar nerve). The scale bar represents 4 mm.

#### AP (Fig. 4)

The AP arise with two heads: an oblique head (oAP) and a transverse head (tAP). Both oAP and tAP are innervated by branches from the deep branch of the ulnar nerve. The oAP branch is thicker than the tAP branch. The oAP branch divides into two main branches after entry. One courses to the ulnar side and divides into two major branches at the origin. One passes deeper to the tAP and innervates the proximal part. The other travels to the insertion of the oAP. During its pathway through the oAP, the major branch sub-divides into many minor branches ramified like a tree. The other main branch curves to the origin of oAP, and continues as a major branch. These major branches pass through the middle part of the muscle to reach the radial part and muscle insertion. During their course through the muscle, these branches sub-divide into many minor branches to innervate 3/5 part of oAP. O-like anastomoses are observed between the major nerve branches. The tAP branch courses to the middle part of tAP after entry.

**Figure 4:**
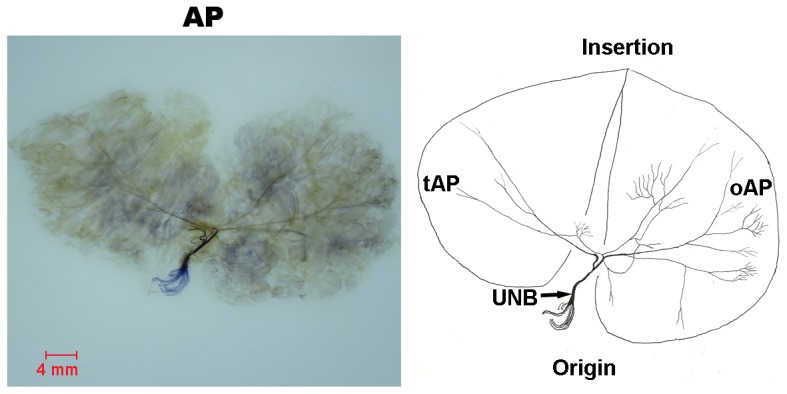
Intramuscular nerve distribution in the AP (right, deep side), drawing demonstrating the oAP and tAP were supplied by the branch of the ulnar nerve (UNB, branch of the ulnar nerve). The scale bar represents 4 mm.

#### ADM (Fig. 5)

The ADM receives innervation from branches of the ulnar nerve. Two main branches accompany each other through the proximal 1/3 of the muscle and then split to the ulnar and radial branches. The ulnar branch innervates 1/3 of the muscle on ulnar side and the radial branch innervates 2/3 of the muscle on radial side. During the ulnar branch’s course to the ulnar part, the main branch divides into major branches densely distributed in the middle part of the muscle with many neural anastomoses.

**Figure 5:**
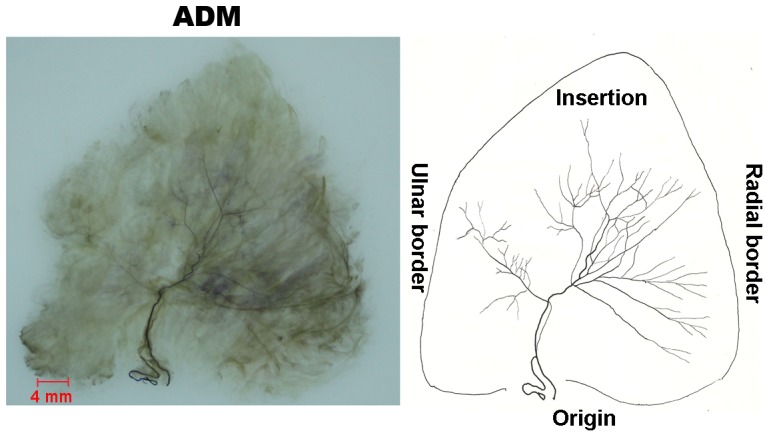
Intramuscular nerve distribution in the ADM (right, deep side), drawing demonstrating the branching pattern of the branch of the ulnar nerve. The scale bar represents 4 mm.

#### FDMB (Fig. 6)

The FDMB is supplied by branches of the ulnar nerve. Two main nerve branches course in parallel to each other and pass through the middle part. Their terminal branches reach the ulnar portion of the muscle. During their course through the muscle, these branches divide into major branches to the radial side. Although the ulnar main branch is thinner, it divides into two or three major branches which anastomose with the radial main branch to make an H-like loop.

**Figure 6:**
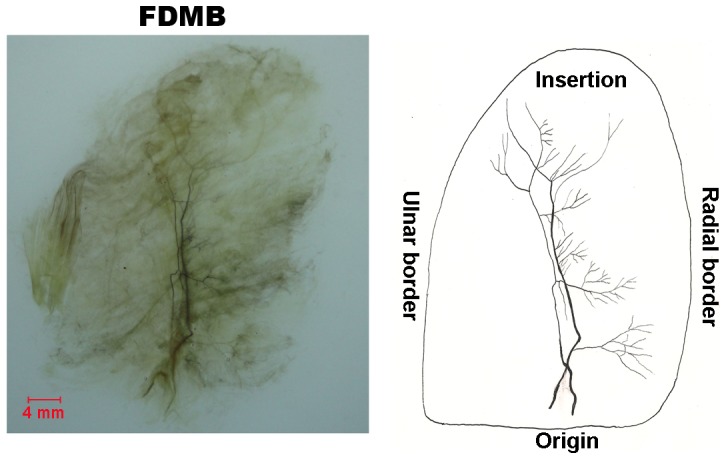
Intramuscular nerve distribution in the FDMB (right, deep side), drawing demonstrating the branching pattern of the branch of the ulnar nerve. The scale bar represents 4 mm.

#### ODM (Fig. 7)

The ODM is innervated by a branch of the ulnar nerve, which enters the muscle from the ulnar side. The main branch passes through the muscle from the origin to the insertion. During its course through the middle part of muscle, the main branch divides into radial major branches and ulnar major branches. These major branches are densely distributed in the middle and distal parts of the muscle belly. The ulnar major branches sub-divide into many minor branches with Y-like and O-like appearances. In addition, two radial major branches anastomose with each other to make a large U-like loop.

**Figure 7:**
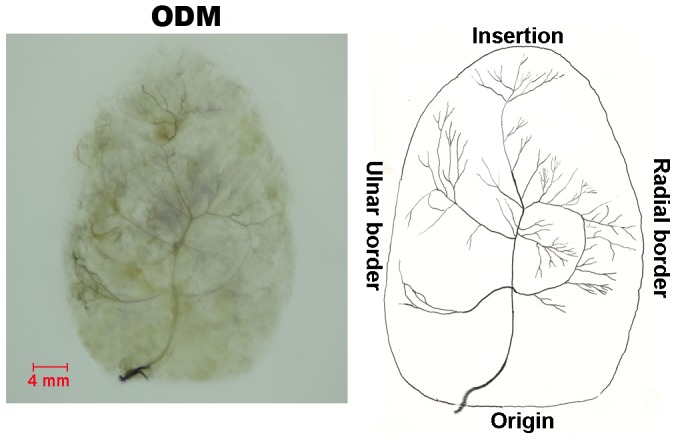
Intramuscular nerve distribution in the ODM (right, deep side), drawing demonstrating the branching pattern of the branch of the ulnar nerve. The scale bar represents 4 mm.

### Relative Spindle Abundance

**Figure 8:**
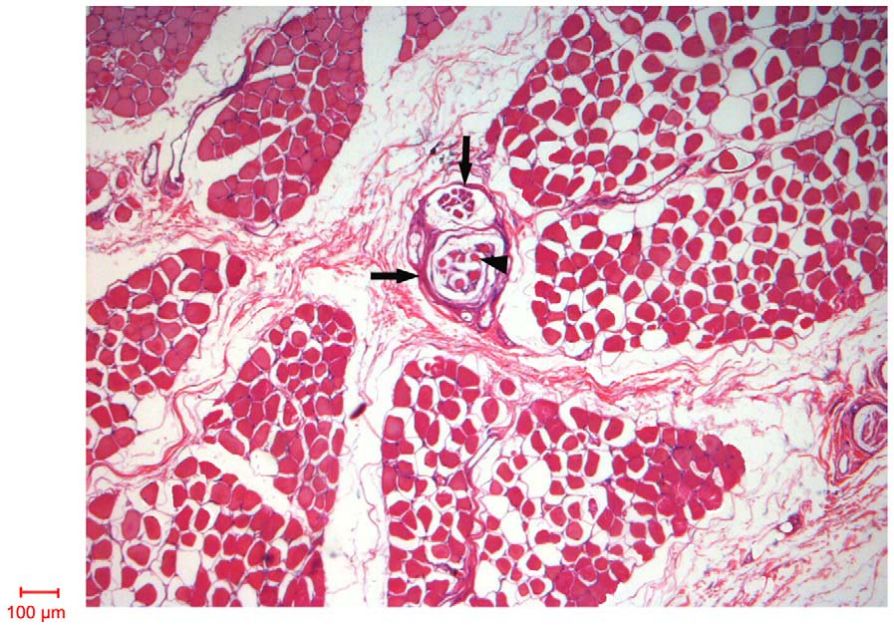
Light microscopic views of muscle spindles in the OP. Transverse section of the OP showing 2 spindles (arrow) arranged side-by-side and forming a paired complex. Their outer capsules are fused but their inner contents remain separate distinct. Each spindle contains several intrafusal fibers (arrowheads). The scale bar represents 100 µm.

Specimen sections were stained using haematoxylin and eosin (H & E) and then examined, whereas equidistant intermediate sections were retained unstained. The microscopic image in Fig.8 demonstrates that muscle spindles are discretely located between muscle bundles in specimen sections. The spindle has a prominent connective tissue capsule enclosing the periaxial space and the intrafusal muscle fibers. Paired spindles were enclosed by a capsule in both the OP and the ODM with each muscle containing two to three paired spindles. There were no tandem arrangements found in these muscles. The relative spindle abundance of the thenar muscles is the following: OP 0.65, FPB 0.62, APB 0.56, and AP 0.52. In the hypothenar muscles, the relative spindle abundance is: ODM 0.59, FDMB 0.51, and ADM 0.40 (Table 1). Furthermore, the relative spindle abundance in the tAP, oAP, superficial head of flexor pollicis brevis (sFPB), and deep head flexor pollicis brevis (dFPB) are 0.34, 0.39, 0.42, and 0.45 respectively. Numerically, the relative spindle abundance of the thenar muscles is greater than that of the hypothenar muscles. The relative spindle abundance of the opponens muscles (OP 0.65 and the ODM 0.59) are the greatest with OP significantly greater than ODM.

## Discussion

The present study clearly demonstrates the details of the intramuscular nerve distribution patterns and the relative spindle abundance in the thenar and hypothenar muscles, which are important to hand surgeons and anatomists.

### APB

The intramuscular minor nerve branches arise from the main branch at the fusion point of proximal third and middle third especially the medial and lateral border of the muscle. In addition, the relative spindle abundance of the APB is greater in our study. These results suggest that if we transplant the muscle in reconstructive surgery, it is very important not to disrupt the continuity of the APB. It should be kept intact because of this intramuscular nerve distribution pattern. On the other hand, the extramuscular nerve trunk of this muscle is the longest among the thenar and hypothenar muscles. Such a long nerve trunk may provide sufficient space for surgeons to use the muscle for transplant.

### FPB

We found that the sFPB is supplied by the recurrent branch of the median nerve and the dFPB is supplied by the deep branch of the ulnar nerve. This muscle could be divided into two compartments; each one is supplied by an independent nerve branch. Each compartment has its own muscle fiber direction and unique sites of origins and endings that distinguishes it from the other compartment. Each compartment has an independent function that can be separated as a flap in surgery [18]. The main nerve was divided into many minor branches after entering the superficial and deep heads; these minor branches were densely distributed at the middle area of the two heads, if we transfer each one of the heads in reconstructive operation, it could obtain good muscle contraction. The FPB plays an important role for the fine movement of the hand because it has the greater relative spindle abundance that is of clinical significance.

### OP

We found that OP is supplied by two independent nerve branches, and there are no connective tissue septations in between. Therefore, this muscle cannot be divided into two muscle compartments. A variety of neural anastomoses were observed in the muscle including the Y-like, H-like, U-like and O-like appearance. The relative spindle abundance of the OP is the greatest among the intrinsic and extrinsic muscles of the hand. We propose that the opposition and the fine movement of the thumb depend on the relative spindle abundance and the neural anastomose patterns of the OP. This muscle, however, has been hardly used as a donor due to its deep location, neural anastomose patterns and greatest relative spindle abundance.

### AP

The AP is constantly innervated by the branches from the deep branch of the ulnar nerve. As compared with the oAP, the tAP’s intramuscular nerve distribution is relatively sparse and the relative spindle abundance is smaller. These findings suggested that the tAP plays fewer roles than the oAP on the fine movements of the hand and maintaining joint stability. The AP has unique anatomical characteristics for its deep location, large muscle mass, and abundant blood supply. It is the muscle that provides powerful grasp and fine pinch for the thumb [19]. We suggest that it should be given much more consideration for the oAP during operations involving AP in hand injuries.

### ADM

The present study demonstrates that the ADM is supplied by two independent nerve branches innervating the ulnar and radial parts, respectively. There are connective tissue septations observed in the muscle. Therefore, the ADM can be divided into the ulnar and radial muscle compartments. The ulnar compartment is smaller compared to the radial compartment which only accounts for one third of the ADM. The major branches of the intramuscular nerve were densely distributed in the middle part of each compartment. We suggest that the two muscle compartments with independent functions be separated as a flap in reconstructive surgery but the independent nerve branch should be intensively protected. The relative spindle abundance of the ADM is the smallest among hypothenar muscles, therefore, it could play an important role in determining the strength of abduction.

### FDMB

Muscle fibers pass obliquely from the ulnar part of the origin to the radial part of the insertion. The intramuscular nerve fibers responsible for innervation of the muscle fibers passed through the middle part of the muscle fibers. Although the muscle is supplied by two independent nerve branches, there are no connective tissue septations observed in between. Therefore, this muscle cannot be divided into muscle compartments. The relative spindle abundance of FDMB is great. Flexion function of the digitus minimus manus can be suitably compensated by the flexor digitorum sublimis and the flexor disitorum profundus [20]. Based on its intramuscular nerve distribution patterns, our results explain why the flexor digiti minimi is transferred as a whole muscle.

### ODM

The intramuscular main branch divides into the radial and ulnar major branches and many neural anastomoses between the branches are Y-like, O-like and U-like in appearance. U-like neural anastomoses may be the characteristic of the ODM and may influence the little finger opposition. The relative spindle abundance the greatest in the hypothenar muscles, and could play an important role in little finger opposition which allows for prehension. The muscle, however, has been hardly served as a donor due to its deep location, intramuscular nerve distribution patterns, and relative spindle abundance.

In summary, this research clearly shows the intramuscular nerve distribution patterns and relative spindle abundance of the thenar and hypothenar muscles in human hand, which would provide important information for athletic physiology of the hand and hand surgery.
